# Impact of the Medicare Benefits Schedule Rebate (MBSR) freeze on General Practice (GP) use: multivariable regression analysis

**DOI:** 10.1186/s12913-023-09569-3

**Published:** 2023-06-07

**Authors:** Shalika Bohingamu Mudiyanselage, Sithara Wanni Arachchige Dona, Julie Abimanyi-Ochom, Jennifer J Watts

**Affiliations:** School of Health and Social Development and Deakin Health Economics, Institute for Health Transformation, Deakin University, Geelong, Australia

**Keywords:** MBS freeze, MBS rebate, General Practice, GP visits, Primary healthcare, Medicare Benefits Schedule

## Abstract

**Background:**

In 2015, the Australian government froze the Medicare Benefits Schedule Rebate (MBSR) for General Practitioner (GP) service use. This paper aimed to explore the impact of the MBSR freeze on the demand for GP services in Victoria, Australia, for three years, from 2014 to 2016.

**Method:**

Annual data on GP service utilisation by the Victorian State Statistical Area Level 3 (SA3) were analysed using 2015 as the reference year (MBSR freeze year). We compared annual per-person GP service use before and after the MBSR freeze for each SA3. Socioeconomic Indexes for Areas (SEIFA) scores and regions of Victoria (Greater Melbourne and the Rest of Victoria) were used to identify the most disadvantaged SA3s in Victoria. We conducted a multivariable regression analysis for the number of GP services per patient by SA3, controlling for regions of Victoria, the number of GP services, the proportion of bulk-billed visits, age group, gender and year.

**Findings:**

After adjusting for age group, gender, region, SEIFA, the number of GPs and the proportion of bulk-billed GP visits, mean GP services per person per year declined steadily between 2014 and 2016, with a 3% or 0.11 visit (-0.114, 95%CI: -0.134; -0.094, P = < 0.001) reduction in mean utilisation in 2016 compared to 2014. In disadvantaged SA3s, there was a fall in the number of GP services that were bulk-billed during and after the MBSR freeze compared to 2014, and this fall was large in LOW SEIFA SA3s, with a reduction in 17% of mean bulk-billed GP services.

**Conclusion:**

The MBSR freeze for GP consultations in 2015 resulted in a reduction in the annual per capita demand for GP visits, with the impact of reduced demand more significant in lower socioeconomic and regional/rural areas. The GP funding policies must consider the demand differences by social-economic status and location.

**Supplementary Information:**

The online version contains supplementary material available at 10.1186/s12913-023-09569-3.

## Background

The supply and demand of primary healthcare in any country vary over time depending on the factors that impact the healthcare market, such as changes in population demographics (including location), government policy (especially policy that affects price), primary healthcare workforce, attitudes of primary care physicians, patients’ individuality and technology [1].

In the Australian healthcare system, private service providers (whether large corporate-owned or locally-owned practices) provide most primary healthcare services, known as General Practitioner (GP) services. GP services are subsidised under the Australian universal healthcare insurance scheme, “Medicare” [2]. The provider sets the service fee, including whether to charge the patient the subsidised fee (with no patient co-payment), where the price the practice receives is equivalent to the rebate under the Medicare Benefits Schedule (MBS). Alternatively, GP practices can charge patients a price premium set above the Medicare Benefits Schedule Rebate (MBSR) (MBSR in 2014-15 was $37.05) [3], in which case the patient co-payment is the difference between the rebate and the price charged by the practice [4, 5].

MBSRs are usually adjusted annually according to the Composite Inflation Index, which frequently is lower than the Consumer Price index [6, 7]. In the past, the GP MBSR was raised annually (MBSR in 2012-13; $ 35.60, in 2013-14; $36.30 in 2014-15; $37.05) following the Government’s Wage Price Index, and it was adjusted to recognise the impact of inflation on GP service running costs [1]. However, in July 2014-15 the Australian government froze the MBSR (at $37.05) as a budget savings plan until July 2018 [8]. To ensure that the length of the GP visit did not reduce with the MBSR freeze, there was a 10 min minimum period per GP visit. In addition, for non-concessional patients, the schedule fee was reduced by $5 to encourage GPs to charge a $5 co-payment from patients to recover the MBS fee reduction [8].

In 2014-15 the predicted impact of the pricing policy was a reduction in income to GPs of 10.6% (from the combined effect of the freeze on rebates and the decline in demand for services as a consequence of the price shift to non-concessional consumers) [8]. The impact on GP income was predicted to be higher again by 2017-18 [9]. To recover the income, it was expected that GPs would charge, on average, a $7-$8 co-payment in 2015-16, increasing to a $12-$15 co-payment in 2017-18 [8]. In addition, it was predicted that GPs would recoup losses by reducing the proportion of services with no co-payment [8]. The impact of a reduction of bulk-billed services and a higher out-of-pocket co-payment is likely to create a barrier for patients and impact their access to primary healthcare. The impact is expected to be most significant in areas of socioeconomic disadvantage.

The purpose of this paper is to explore the impact of the 2014-15 MBSR freeze on the demand for GP service use in Victoria, Australia, from 2013-14 to 2015-16 by the local area and to determine if there was a difference by geographic, and socioeconomic disadvantage.

## Method

### Data source and population sample

Annual GP service data were requested from the Australian Government Department of Human Services for the years 2013-14, 2014-15 and 2015-16 by Statistical Area Level 3 (SA3) (geographic area with population size 30,000-130,000) [10] for Victoria, Australia. The data requested were for patient claims for GP services for each of the three years based on the patient’s location (by SA3). Service claims were for GP services under MBS Item 23 for professional attendance by a general practitioner lasting less than 20 min at the GP clinic.

### Variables

The requested data for each SA3 included the total number of GP services claimed by year, age groups (0–14, 15–39, 40–64, 65+) and gender with additional variables for the number of services with no co-payment, total benefits paid under the MBS, total out-of-pocket charges, and the total cost of the GP services (claimed and out-of-pocket). Later collected GP claims were merged with the total population numbers (collected from the Australian Bureau of Statistics, ABS) for each SA3 by year, gender, age group and Socioeconomic Indexes for Areas (SEIFA) [11]. GP visits per capita were calculated using population numbers per each SA3. The SEIFA code provides a series of indicators of relative economic disadvantage for a geographic region. For this analysis, the SEIFA code used to identify the most and least disadvantaged SA3s was the Index of Relative Socioeconomic Advantage and Disadvantage (IRSAD). Australia’s current average IRSAD score is 1000, with a standard deviation of 100 [12]. Referencing this, we categorised the Victorian SA3s into three main sub-groups: “LOW SEIFA” IRSAD score of less than 950, “MID SEIFA” as IRSAD score between 951 and 1050 and “HIGH SEIFA” IRSAD score of more than 1051 [11]. We collected the total number of GPs at each SA3 from the Health Workforce Australia data tool [13] for each SA3 for 2014, 2015 and 2016. We also categorise each SA3 by their region as the “Greater Melbourne area” or the “Rest of Victoria” (using the ABS geographical classification) as a proxy for access to GP service providers [14].

### Statistical analysis

The analysis was descriptive and exploratory to determine how the average total GP services claimed had changed over time for LOW, MID and HIGH SEIFA SA3s by age group and gender before and after the MBSR freeze. The number of GPs, bulk-billed proportion, age group, gender, Victorian region and SEIFA category were treated as covariates. We defined the most disadvantaged group for our study population as represented by people who lived in LOW or MID SEIFA SA3s and who were not living in the greater Melbourne area. Twenty-five disadvantaged SA3s were included using this definition. We used a multivariable regression equation with the annual average number of GP visits claimed per patient as the dependent variable to determine the predictors of GP service claims controlling for the year (as a fixed effect), age group and gender. Explanatory variables included in the regression model were region (greater Melbourne or the Rest of Victoria), the number of GPs and the proportion of services without a co-payment. The model included dummy variables for the year for observed time effects and SEIFA for socioeconomic differences at SA3 level. In addition, we clustered the standard errors at the area level (SA3) to account for potential unobserved variation.

We then undertook a backward stepwise regression [14] to explore the most influential factors (variables) for GP service use with adjustment for the number of GPs, bulk-billed proportion, age group, gender, Victorian region and SEIFA category. This model is referred to as the adjusted model.

The dollar values presented in this paper are in Australian dollars. The year format of this paper is referred to the Australian financial year format, in which a year starts on July 1st and ends the next year on June 30th ; for example, if we have mentioned years as “2014” or “2013-14”, the year starts from July 1^st^ of 2013 and ends on June 30^th^ of 2014.

## Results

Table 1 shows the population baseline characteristics, age group, gender, and number of GPs by LOW, MID and HIGH SEIFA categories for Victoria. There are 65 SA3s in Victoria, with the majority classified in the MID SEIFA category (48%). There are no HIGH SEIFA SA3s outside the greater Melbourne area. The higher number of GPs are in MID SEIFA SA3s, and the per capita rate of GPs is highest in LOW SEIFA SA3s. We identified 45 disadvantaged SA3s by SEIFA category (LOW;14 and MID;31), and 25 of the disadvantaged SEIFAs were in regional Victoria (Rest of Victoria) (Table 1).

**Table 1:** Baseline characteristics (2013–2014)

	LOW SEIFA	MID SEIFA	HIGH SEIFA
**Total SA3s, n (%)**	14(22%)	31(48%)	20(30%)
*Greater Melbourne*	*3(21%)*	*17(55%)*	*20(100%)*
*Rest of Victoria*	*11(79%)*	*14(45%)*	*0(0%)*
**Total population %**	17%	51%	32%
*0–14 years*	*19%*	*20%*	*16%*
*15–39 years*	*34%*	*34%*	*39%*
*40–64 years*	*32%*	*32%*	*31%*
*65 + years*	*16%*	*14%*	*15%*
Gender (male, %)	*22%*	*48%*	*31%*
Mean GPs per 10,000 population (mean, 95%CI)	8.05(7.08:9.03)	11.27(10.31:12.23)	12.67(15.52:13.83)
Mean GP visits (mean, SD)	4.32 ± 1.73	4.20 ± 1.66	3.63 ± 1.44

GP General Practitioner, SEIFA Socio-Economic Indexes for Areas, SD Standard Deviation, SA3 Statistical Area Level 3

Table 2 shows the mean number of GP visits per person yearly before, during and after the freeze. There is a non-statistically significant decline in annual GP visits per person in all LOW, MID and HIGH SEIFAs. The utilisation pattern by age group shows that GP visits per person per year fell in MID and HIGH SEIFA SA3s in all age groups. For LOW SEIFA SA3s, a fall in annual GP service use was found in the 40 + year age groups. For services that were bulk-billed (i.e. where there was no patient co-payment), LOW SEIFA SA3s were found to have a higher proportion of services bulk-billed. There was a reduction in the proportion of services bulk-billed for the age group 65 + years after the freeze, while other age groups had an increase in the proportion of services bulk-billed over the 2014–2016 period.

**Table 2:** GP service use by SEIFA category before, during and after the MBSR Freeze

	Before Freeze mean ± SD	During Freeze mean ± SD	After Freeze mean ± SD	Mean difference, 95%CI (During-Before)	Mean difference, 95%CI (During-After)
**LOW SEIFA**					
**Number of GP visits per person per year**	4.32 ± 1.73	4.30 ± 1.67	4.28 ± 1.63	-0.02(-0.46;0.43)	-0.03(-0.48;0.41)
*0–14 years*	*2.70 ± 0.56*	*2.74 ± 0.54*	*2.71 ± 0.49*	*0.04(-0.25;0.32)*	*0.01(-0.27;0.29)*
*15–39 years*	*3.50 ± 1.07*	*3.52 ± 1.09*	*3.57 ± 0.46*	*0.03(-0.55;0.61)*	*0.07(-0.51;0.65)*
*40–64 years*	*4.35 ± 0.90*	*4.32 ± 0.86*	*4.32 ± 0.81*	*-0.03(-0.50;0.44)*	*-0.03(-0.49;0.43)*
*65 + years*	*6.73 ± 0.76*	*6.63 ± 0.78*	*6.54 ± 0.71*	*-0.10(-0.51;0.32)*	*-0.18(-0.58;0.21)*
**Percentage of bulk-billed GP visits**	**85.12 ± 10.18**	**86.17 ± 6.62**	**86.57 ± 9.25**	**1.05(-1.56;3.66)**	**1.45(-1.12;4.01)**
*0–14 years*	*91.59 ± 6.16*	*93.17 ± 5.68*	*93.75 ± 5.21*	*1.59(-1.59;4.76)*	*2.17(-0.86; 5.22)*
*15–39 years*	*80.82 ± 8.97*	*82.05 ± 7.92*	*82.81 ± 7.39*	*1.23(-3.30;5.77)*	*1.99(-2.41;6.39)*
*40–64 years*	*76.77 ± 10.26*	*77.91 ± 9.61*	*78.89 ± 9.34*	*1.13(-4.19;6.46)*	*1.81(-3.45;7.07)*
*65 + years*	*91.31 ± 5.30*	*91.57 ± 4.92*	*91.13 ± 5.20*	*0.26(-2.48;3.00)*	*-0.19(-3.00;2.62)*
**MID SEIFA**					
**Number of GP visits per person per year**	**4.20 ± 1.66**	**4.16 ± 1.63**	**4.10 ± 1.59**	**-0.05(-0.34;0.24)**	**-0.10(-0.39;0.18)**
*0–14 years*	*2.93 ± 0.45*	*2.90 ± 0.42*	*2.84 ± 0.42*	*-0.04(-0.19;0.11)*	*-0.10(-0.25;0.06)*
*15–39 years*	*3.26 ± 1.03*	*3.25 ± 1.04*	*3.25 ± 1.04*	*-0.01(-0.37;0.37)*	*-0.01(-0.38;0.36)*
*40–64 years*	*4.10 ± 0.84*	*4.04 ± 0.82*	*4.00 ± 0.79*	*-0.06(-0.36;0.23)*	*-0.09(-0.38;0.20)*
*65 + years*	*6.52 ± 1.05*	*6.43 ± 1.05*	*6.31 ± 1.04*	*-0.09(-0.47;0.28)*	*-0.21(-0.58;0.16)*
**Percentage of bulk-billed GP visits**	**81.05 ± 11.80**	**81.62 ± 11.45**	**82.29 ± 10.81**	**0.57(-1.48;2.62)**	**1.23(-0.76;3.23)**
*0–14 years*	*87.09 ± 8.15*	*88.05 ± 7.73*	*89.04 ± 7.19*	*0.96(-1.86;3.78)*	*1.96(-0.77;4.69)*
*15–39 years*	*77.43 ± 8.54*	*78.14 ± 8.19*	*79.04 ± 7.60*	*0.71(-2.26;3.68)*	*1.61(-1.26;4.48)*
*40–64 years*	*71.81 ± 11.17*	*72.38 ± 10.79*	*73.23 ± 10.03*	*0.57(-3.33;4.48)*	*1.42(-2.36;5.19)*
*65 + years*	*87.89 ± 10.71*	*87.93 ± 10.32*	*87.85 ± 9.55*	*0.04(-3.70;3.78)*	*-0.05(-3.66;3.56)*
**HIGH SEIFA**					
**Number of GP visits per person per year**	**3.63 ± 1.44**	**3.57 ± 1.41**	**3.52 ± 1.37**	**-0.06(-0.38;0.25)**	**-0.11(-0.42;0.20)**
*0–14 years*	*2.79 ± 0.27*	*2.75 ± 0.25*	*2.69 ± 0.25*	*-0.05(-0.16;0.07)*	*-0.10(-0.22;0.01)*
*15–39 years*	*2.50 ± 0.80*	*2.46 ± 0.79*	*2.46 ± 0.80*	*-0.04(-0.39;0.32)*	*-0.05(-0.40;0.31)*
*40–64 years*	*3.46 ± 0.59*	*3.41 ± 0.56*	*3.38 ± 0.53*	*-0.05(-0.31;0.20)*	*-0.08(-0.33;0.17)*
*65 + years*	*5.76 ± 0.84*	*5.65 ± 0.80*	*5.54 ± 0.76*	*-0.12(-0.47;0.26)*	*-0.23(-0.58;0.13)*
**Percentage of bulk-billed GP visits**	**69.69 ± 12.87**	**69.99 ± 12.73**	**70.31 ± 12.55**	**0.30(-2.52;3.11)**	**0.62(-2.18;3.42)**
*0–14 years*	*71.94 ± 11.72*	*72.97 ± 12.03*	*73.67 ± 12.81*	*1.03(-4.26;6.31)*	*1.73(-3.74;7.19)*
*15–39 years*	*65.19 ± 10.18*	*65.79 ± 10.00*	*66.85 ± 9.45*	*0.60(-3.90;5.09)*	*1.66(-2.71;6.03)*
*40–64 years*	*61.04 ± 10.43*	*61.34 ± 10.26*	*61.81 ± 9.94*	*0.31(-4.30;4.91)*	*0.77(-3.76;5.31)*
*65 + years*	*80.60 ± 10.05*	*89.87 ± 10.34*	*78.92 ± 10.76*	*-0.73(-5.27;3.81)*	*-1.68(-6.31;2.96)*

GP General Practitioner, SEIFA Socio-Economic Indexes for Areas, SD Standard deviation, MBSR Medicare Benefits Schedule Rebate

The mean number of GP visits and how the mean has changed over the study period for individual SA3s are shown in the additional files (Additional file 1). During the MBSR freeze in 2014-15, there were 43 (66%) SA3s that showed a decrease in overall GP service use compared to 2013-14, and this increased to 75% of SA3s after the MBSR Freeze in 2015-16 compared to 2014-15.

### GP service use in most disadvantaged SA3s before, during and after the MBSR freeze

In disadvantaged SA3s, there was a fall in the number of GP services that were bulk-billed during and after the MBSR freeze compared to 2013-14 (Table 3), and this fall was greater in LOW SEIFA SA3s, with a reduction of 17% of mean bulk-billed GP services. In disadvantaged SA3s, the proportion of total GP services that were bulk-billed increased during and after the freeze, but the mean number of GP visits per person per year fell during the freeze. The mean number of GP visits per person per year in LOW SEIFA SA3s increased after the freeze to be higher than before the freeze, but this was not the case for MID SEIFA SA3s.

**Table 3:** GP service use characteristics for disadvantaged SA3s

	Before Freeze		During Freeze			After Freeze		Mean difference, 95%CI (During- before)		Mean difference, 95%CI (During- after)	
	Low SEIFA	Mid SEIFA	Low SEIFA	Mid SEIFA	Low SEIFA		Mid SEIFA	Low SEIFA	Mid SEIFA	Low SEIFA	Mid SEIFA
Number of SA3s, n(%)	14(22%)	31(48%)	14(22%)	31(48%)	14(22%)		31(48%)	-	-		
*Population with a bulk-billed service (%)	38%	62%	22%	55%	21%		55%	-17%	-7%	-1%	0%
**Proportion of total GP services that are bulk-billed, %±SD	85.12 ± 10.18	81.05 ± 11.80	86.17 ± 6.62	81.62 ± 11.45	86.57 ± 9.25		82.29 ± 10.81	1.05(-1.56;3.66)	0.57(-1.48;2.62)	1.45(-1.12;4.01)	1.23(-0.76;3.23)
Mean GP visits per year per person, mean ± SD	4.32 ± 1.73	4.20 ± 1.66	4.30 ± 1.67	4.16 ± 1.63	4.28 ± 1.63		4.10 ± 1.59	-0.02(-0.46;0.43)	0.05(-0.34;0.24)	-0.03(-0.48;0.41)	-0.10(-0.39;0.18)

GP General Practitioner, SEIFA Socio-Economic Indexes for Areas, SD Standard deviation, SA3 Statistical Area Level 3, *percentage of the population who had bulk-billed GP service; ** proportion of bulk-billed GP service from total GP services

Table 4 shows the multivariable regression results for the mean number of GP visits per person per year by SA3 before, during and after the MBSR freeze controlling for confounders. After adjusting for age group, gender, the region in Victoria, SEIFA, the number of GPs and the proportion of bulk-billed visits, GP service use declined steadily between 2013-14 and 2015-16, with a 3%, 0.11 visits reduction in utilisation in 2015-16 (-0.114, 95%CI: -0.134; -0.094, P < 0.001) compared to 2013-14 (Table 4). The coefficient for the proportion of bulk-billed services is positive and significant, indicating that GP visits increased by 0.023 (95%CI: 0.021; 0.024, P = < 0.001) for each 1% increase in the proportion of bulk-billed (Table 4).

**Table 4:** The impact of MBSR Freeze on annual GP visits, multivariable analysis results

Explanatory variables	Dependent variable: Mean GP visits per person per year		
	Coefficient	95% CI	p-value
**Age group**			
*0–14 years*	Reference		
*15–39 years*	0.475	0.034: 0.917	< 0.001
*40–64 years*	1.431	1.059: 1.802	< 0.001
*65 + years*	3.384	2.910: 3.858	< 0.001
**Gender**			
*Female*	Reference		
*Male*	-0.712	-0.833: -0.591	< 0.001
**Region in Victoria**			
Greater Melbourne	Reference		
Rest of Victoria	-0.184	-0.368: -0.001	< 0.001
**SEIFA category**			
*HIGH*	Reference		
*LOW*	0.474	0.340: 0.607	< 0.001
*MID*	0.380	0.314: 0.447	< 0.001
**Year**			
*2013-14*	Reference		
*2014-15*	-0.058	-0.082: -0.035	< 0.001
*2015-16*	-0.114	-0.134: -0.094	< 0.001
**Number of GPs in SA3**	-0.001	-0.003: 0.001	0.498
**Bulk-billed Proportion**	0.023	0.021: 0.024	< 0.001

n = 1,560, R^2^ = 0.8159, F(11, 1548) = 623.58, Cook-Weisberg test for heteroskedasticity < 0.001, vce corr test for multicolineriarty did not show signs for correlation across variables

GP General Practitioner, CI Confidence Interval, SA3 Statistical Area Level 3, MBSR Medicare Benefits Schedule Rebate

## Discussion

Healthcare price changes are important since they change the behaviour of the principal parties in healthcare markets, such as patients, health professionals, healthcare providers and health systems. Freezing annual increases in GP MBSR and frozen MBSR for four straight years in a competitive healthcare market would be expected to be passed on to the patients by the GPs. The potential increase in out-of-pocket payments to patients would reduce the use of services, in this case, GP services. This reduction would also affect the revenue available to GPs from service delivery (ceteris paribus law– no GP change in behaviour), conditional on how demand for GP services responds to the price change.

In reality, the healthcare market is not competitive as government intervention is significant and impacts both the supply and demand of GP services, leading to different outcomes in the responsiveness of demand and supply to price changes. There is limited recent evidence in Australia of the responsiveness of demand to price changes [15], more so the impact of price changes across the population. The price elasticity of demand for GP services is expected [16] to be slightly inelastic – the results highlight that, in general, across all SA3s, the average GP service use did not change significantly over the freeze years (Table 2).

Another factor that will impact GP price changes is an increase in the costs of service delivery, such as GP practice staff costs, medical products costs, utilities and other costs. An increase in practice costs is likely to result in GPs passing on these increased costs to patients. If the government rebate remains the same, this would be expected to increase the out-of-pocket patient charges. Alternatively, GPs can reduce the proportion of bulk-billed services. High out-of-pocket costs and/or a reduction in bulk-billed services will reduce demand for GPs, with patients deferring visits until their condition worsens or choosing to seek care elsewhere [17], for example, hospital Emergency Departments (EDs).

The adjusted model showed that the MBSR freeze did result in a 3% reduction in the annual per capita demand for GP visits between 2014 and 2016 after controlling for all confounders. We do not know the impact on GP revenue over the same timeframe, so GP revenue may have declined over the same period. The coefficient on the proportion of services that were bulk-billed was positive but small, suggesting that the annual GP visits increased by 0.02 for each 1% increase in the proportion of bulk-billing from 2014 to 2016 (Table 4). The results in the unadjusted models, specifically looking at socioeconomic disadvantage and location, indicate that the impact of the freeze, in terms of the mean number of services per person and the bulk-billing rate, differed across population groups.

Considering the level of socioeconomic disadvantage, there was a decline in GP visits per person per year of 0.03 for LOW SEIFA SA3s compared to 0.1 and 0.11 for MID and HIGH SEIFA SA3s, after the freeze (Table 2). In addition, for socioeconomic disadvantaged SA3s (MID and LOW SEIFA), there was a reduction in the mean percentage of the population with a bulk billed GP service during the MBSR freeze (2014 to 2016), a decline of 17% for LOW SEIFA and 7% for MID SEIFA SA3s (Table 3).

Subsidised GP services have been shown to improve access to primary care for socioeconomically disadvantaged groups, and bulk billing is an additional policy in Australia that supports access to GP services [18]. The implication of a reduction in bulk-billed services is an increase in GP services that charge out-of-pocket (holding all else constant) hence an increase in the price consumers face to access GP services [19]. The impact on demand will be different across population groups; socioeconomically disadvantaged populations have been shown to be more price sensitive [20–22]. Hence a price increment may be one explanation for the reduced demand for health care observed during the MBSR freeze [17]. Reduced healthcare demand in socioeconomically disadvantaged populations has been shown to lead to worse health outcomes – in that patients are likely to present to the GP or hospital after their condition has worsened, leading to higher healthcare costs in the long run [17]. Generally, low socioeconomic disadvantaged populations have been shown to be at a greater risk of poor health and a higher prevalence of illness [23, 24]. Similarly, socioeconomically disadvantaged neighbourhoods have been shown to reduce the odds of having a usual source of care, limiting access to preventive services and leading to increased unmet medical needs [25]. There is also evidence of per capita rate of medical practitioners decreasing with increasing levels of relative disadvantage [24, 26].

Regression analysis controls for other confounders in addition to SEIFA and freeze years. Results show a reduction of GP service use in the Rest of Victoria compared to Greater Melbourne, indicating reduced demand compared to Greater Melbourne. High competition in the GP market in the greater Melbourne area implies that it is difficult for practitioners to increase the price since consumers have a variety of alternative GPs to choose from, hence demand was higher relative to the Rest of Victoria. On the contrary, for the Rest of Victoria, limited competition implies that price increments through out-of-pocket payments may be possible, and hence demand responds by falling/decreasing. This may be a result of patients seeking care elsewhere due to extra out-of-pocket costs, for example, emergency departments.

Average GP service use per patient was higher for socioeconomically disadvantaged populations (LOW and MID SEIFA SA3s compared to HIGH SEIFA SA3s). Studies have shown that people living in disadvantaged areas are more likely to consult their GP than those in less disadvantaged areas. This may be an indication of poorer health for socioeconomically disadvantaged populations relative to less disadvantaged populations [23, 24].

Strengths of this study include real data retrieved from Australian Government Department of Human Services that represents actual utilisation of GP services by SA3 regions in Victoria, one of the most densely populated states in Australia, over three years before, during and after major policy change that resulted in a freeze in the MBS rebate to GPs. The data were pre-Covid pandemic hence revealing a response in the market where the only difference was that of the price freeze. Limitations include that the data relate to a single Medicare Item number for GP services only and do not include other services that may have been used instead (e.g. ED, other primary care practitioners or medical specialists, or other GP Item numbers); Victoria is densely populated therefore results may not be reflective of remote Australia, and we were not able to split into both rural and larger regional area. Although SEIFA was chosen as a proxy for socioeconomic disadvantage, it is not exhaustively reflective of the wealth of SA3s, it does not include other indicators like employment/unemployment, income separately; deconstructed SEIFA components may be a better indicator of how these impact the average GP service use per person especially in response to price change [11].

## Conclusion

The Medicare Benefits Schedule Rebate freeze resulted in a reduction in the annual per capita demand for GP visits between 2014 and 2016, and this reduction was accentuated in the most disadvantaged populations. Further, we recommend researchers to investigate the 2015 GP MBSR freeze impact on all States and Territories in Australia and also recommend policymakers to consider the demand differences by social-economic status and location when developing new funding policies on primary healthcare.

## Electronic supplementary material

Below is the link to the electronic supplementary material.

Supplementary Material 1

## Data Availability

Limited data sets to interpret the reported study results are available from the corresponding author on reasonable request.

## List of Abbreviations

MBS: Medicare Benefits Schedule
MBSR: Medicare Benefits Schedule Rebate
GP: General practice/practitioner
SA3: Statistical Area Level 3
ED: Emergency Department
