# Supplementary material for: Impact of the Medicare Benefits Schedule Rebate (MBSR) freeze on General Practice (GP) use: multivariable regression analysis
Source: BMC Health Serv Res. 2023 Jun 7;23:588. doi: 10.1186/s12913-023-09569-3 (PMC10249270; doi:10.1186/s12913-023-09569-3)
Supplement: Supplementary file 1 — Supplementary Material 1 [file 12913_2023_9569_MOESM1_ESM.docx]

### Additional files

### Additional file 1: GP service use change (rate) before and after freeze by patient SA3

|  | **Number of patient SA3 (%)** | | | | | | | | |
| --- | --- | --- | --- | --- | --- | --- | --- | --- | --- |
| **Rate %** | **GP service use change 2013-14 to 2014-15** | | | **GP service use change 2014-15 to 2015-16** | | | **GP service use change 2013-14 to 2015-16** | | |
|  | **LOW SEIFA** | **MID SEIFA** | **HIGH SEIFA** | **LOW SEIFA** | **MID SEIFA** | **HIGH SEIFA** | **LOW SEIFA** | **MID SEIFA** | **HIGH SEIFA** |
| **Increased** | **8(57%)** | **12(39%)** | **2(10%)** | **6(43%)** | **9(29%)** | **1(5%)** | **8(57%)** | **9(29%)** | **2(10%)** |
| 1.0 - 2.0% | 6(9%) | 7(11%) | 2(3%) | 3(5%) | 7(11%) | 0(0%) | 3(5%) | 5(8%) | 1(2%) |
| 2.1 - 4.0% | 1(2%) | 3(5%) | 0(0%) | 2(3%) | 0(0%) | 0(0%) | 2(3%) | 3(5%) | 0(0%) |
| 4.1- 7.0% | 1(2%) | 2(3%) | 0(0%) | 0(0%) | 2(3%) | 1(2%) | 1(2%) | 0(0%) | 1(2%) |
| >7.1% | 0(0%) | 0(0%) | 0(0%) | 1(2%) | 0(0%) | 0(0%) | 2(3%) | 1(2%) | 0(0%) |
| **Decreased** | **6(43%)** | **19(61%)** | **18(90%)** | **8(57%)** | **22(71%)** | **19(95%)** | **6(43%)** | **22(71%)** | **18(90%)** |
| 1.0 -2.0% | 3(5%) | 8(12%) | 7(11%) | 4(6%) | 14(22%) | 12(18%) | 1(2%) | 7(11%) | 4(6%) |
| 2.1 - 4.0% | 2(3%) | 8(12%) | 11(17%) | 4(6%) | 6(9%) | 7(11%) | 2(3%) | 6(9%) | 6(9%) |
| 4.1- 7.0% | 1(2%) | 2(3%) | 0(0%) | 0(0%) | 1(2%) | 0(0%) | 1(2%) | 5(8%) | 8(12%) |
| >7.1% | 0(0%) | 1(2%) | 0(0%) | 0(0%) | 1(2%) | 0(0%) | 2(3%) | 4(6%) | 0(0%) |
